# Sperm Phosphoproteome: Unraveling Male Infertility

**DOI:** 10.3390/biology11050659

**Published:** 2022-04-25

**Authors:** Rebeca Serrano, Luis J. Garcia-Marin, Maria J. Bragado

**Affiliations:** Grupo de Investigación en Señalización Intracelular y Tecnología de la Reproducción (SINTREP), Instituto de Investigación INBIO G+C, Universidad de Extremadura, 10003 Caceres, Spain; rebecasp@unex.es (R.S.); ljgarcia@unex.es (L.J.G.-M.)

**Keywords:** human spermatozoa, sperm proteins, PTM, phosphorylation, phosphoproteomics, sperm motility, male infertility

## Abstract

**Simple Summary:**

Approximately 24% of men referred to assisted reproductive technology (ART) present with idiopathic male infertility. The current standard analysis of human semen does not allow for an accurate diagnosis of this infertility with unknown etiology. Spermatozoa cellular development and maturation, as well the acquisition of suitable motility and capacitation, are tightly coordinated by sperm protein phosphorylation, among other protein post-translational modifications. Extraordinary advances have been achieved in the field of spermatozoa using proteomics methodology in combination with bioinformatics. The aim of this work is to review, using a proteomic and phosphoproteomic analysis, the updated knowledge about proteins and phosphoproteins of spermatozoa that regulate cell processes necessary to achieve a proper fertilization. The phosphorylation of sperm proteins involved in spermatogenesis, in sperm capacitation, and in the maintenance of correct sperm motility, and consequently in sperm quality, is focused on in this review. Further investigations of protein spermatozoa in larger populations combined with other multi-omics technologies would offer a precise perspective of male fertility and would be especially relevant for those cases involving repeated failures in ART linked to idiopathic infertility.

**Abstract:**

Infertility affects approximately 15% of couples worldwide of childbearing age, and in many cases the etiology of male infertility is unknown. The current standard evaluation of semen is insufficient to establish an accurate diagnosis. Proteomics techniques, such as phosphoproteomics, applied in this field are a powerful tool to understand the mechanisms that regulate sperm functions such as motility, which is essential for successful fertilization. Among the post-translational modifications of sperm proteins, this review summarizes, from a proteomic perspective, the updated knowledge of protein phosphorylation, in human spermatozoa, as a relevant molecular mechanism involved in the regulation of sperm physiology. Specifically, the role of sperm protein phosphorylation in motility and, consequently, in sperm quality is highlighted. Additionally, through the analysis of published comparative phosphoproteomic studies, some candidate human sperm phosphoproteins associated with low sperm motility are proposed. Despite the remarkable advances in phosphoproteomics technologies, the relatively low number of studies performed in human spermatozoa suggests that phosphoproteomics has not been applied to its full potential in studying male infertility yet. Therefore, further studies will improve the application of this procedure and overcome the limitations, increasing the understanding of regulatory mechanisms underlying protein phosphorylation in sperm motility and, consequently, in male fertility.

## 1. Introduction

Our understanding of sperm physiology remains relatively superficial despite the large number of manuscripts focused on spermatozoa [1]. Indeed, a global estimation calculates that 1 in 15 men of reproductive age are infertile, and the diagnosis of idiopathic male infertility, for which the cause is unknown, is a reality for 24% of men referred to assisted reproductive technology (ART) [2]. To date, basic semen analysis, or seminogram, is the best predictive test used routinely in laboratories for the assessment of male partner fertility, where it is analyzed if the semen samples meet the macroscopic (volume, pH, color, and viscosity) and microscopic characteristics (sperm concentration, total motility, progressive motility, and sperm morphology) established by the World Health Organization (WHO), which published a set of guidelines for evaluating semen quality 40 years ago. The last update, in 2010, includes the current reference parameters to make the first prognosis of male infertility [3].

Nevertheless, the seminogram is not the most suitable analysis to obtain an accurate diagnosis because semen parameters within the reference interval do not guarantee fertility, nor do values outside those limits necessarily imply male infertility or pathology [4]. Several studies have shown that men with sperm parameters (sperm number, morphology, and motility) below the thresholds outlined by the WHO can be fertile [5,6,7,8]. Additionally, in the same way, there are cases of men with normal sperm parameters that are infertile [9]. Therefore, more in-depth analysis and understanding of spermatozoa physiology at the molecular level are necessary to improve the current evaluation of male fertility by the routine semen analysis.

Fertilization might be considered the endpoint of sperm function. To get it successfully, spermatozoon, a highly specialized haploid cell that contains exceptionally condensed chromatin and will deliver the paternal DNA to the oocyte, must be completely functional. For that, spermatozoa undergo a series of physiological and biochemical changes from their developmental stages and during their transit through the male first and female reproductive tract later, which occur apparently in the complete absence of simultaneous gene transcription and protein translation. Although several coding and non-coding RNAs exist in human spermatozoa [10], which may play a role in gene silencing or heterochromatinization, their transcriptional and translational activities are nearly silent. Thus, sperm proteins of mature spermatozoa might undergo different post-translational modifications (PTM), such as phosphorylation or acetylation among, and become very important molecular mechanisms by which spermatozoa acquire functionality [11]. For this reason, the alteration of sperm status (for example, by errors in spermatogenesis or maturation) may be accompanied by a distinctive pattern of PTMs, characteristic of spermatozoa, in particular with low quality or motility and, therefore, low male reproductive prognostic.

In this regard, it is necessary to focus our attention on sperm motile capacity, and the abnormal content or presence of PTMs, such as the reduced abundance of lysine glutarylation in several proteins located in the tail of human spermatozoa, just as a diminished quantity of S-sulfhydrated H3 and H3.3 histones positively correlate with sperm progressive motility [12,13]; conversely the level of SUMO1-positive spermatozoa and the quantity of lysine 2-hydroxyisobutyrylation in sperm proteins trend towards higher grades in asthenozoospermic men compared with normozoospermic ones, indicating a negative association with the motility of human spermatozoa in this case [14,15]. Additionally, lysine acetylation seems to be essential for human sperm motility and fertilization [16].

The most extensively studied PTM in human spermatozoa is phosphorylation [17]. The phosphorylation of specific sperm proteins plays an important role in regulating sperm processes essential for fertilization, such as sperm motility, capacitation, or acrosome reactions [18,19,20]. Extraordinary advances have been achieved in the field of male infertility in recent decades, especially with the use of proteomics techniques and the bioinformatic analysis of human sperm proteomic data. However, there are many well-recognized causes of male infertility in humans whose molecular basis is only just beginning to be understood. The study of the global protein phosphorylation landscape of spermatozoa in different species proposes wide phosphoregulation in other processes such as sperm formation [21,22], maturation [23], capacitation [24,25,26,27,28], and motility [18,29,30,31,32]. Nevertheless, it remains to be fully explained which are ultimately the molecular mechanisms responsible for spermatozoa motility and, therefore, for sperm quality. Advances in global and quantitative methods to elucidate dynamic phosphorylation events in spermatozoa will be essential for a systematic understanding of their functional behavior. They will allow for a more comprehensive analysis of the biochemical basis of defective semen quality and identify possible biomarkers for different pathologies and conditions related to infertility. A few studies applying quantitative mass spectrometry (MS)-based proteomics have proposed some molecular mechanisms through which protein phosphorylation might affect sperm motility in humans [18,29,30,31].

From a proteomic perspective, this review summarizes the current knowledge of protein phosphorylation in human spermatozoa as a molecular mechanism responsible for the regulation of spermatozoa motility, and subsequently of sperm quality.

## 2. Proteomics and Sperm Physiology

According to the recent data, the proteomic approach is a powerful tool to identify human sperm proteins as biomarkers of fertility [33]. For example, a recent seminal plasma proteomic-based study proposes the HSPA2 protein, a molecular chaperone mediating protein folding, as a possible biomarker of spermatogenesis status. Azoospermic men (who have a complete absence of spermatozoa in their ejaculate) lack HSPA2, which is present as additional protein isoforms in cryptozoospermia (<0.1 million spermatozoa mL^−1^) [34]. In addition, a study comparing high- and low-quality sperm nuclear extracts by proteomic analysis recently showed that the presence of Topoisomerase 2A in the human spermatozoa head is highly correlated to poor head morphology. So, Topoisomerase 2A, a protein normally involved in the alteration of DNA topology, may also be considered a potential biomarker to confirm male infertility in clinical practice [35]. The first study that examined the potential variability of the proteome in different semen samples and proposed proteomics as a useful tool for studying defects in sperm function was published almost 2 decades ago by Pixton and collaborators [36]. Since then, many proteomics studies have performed a comparative proteomics analysis between sperm cells from infertile patients and healthy donors [37,38,39,40,41,42,43,44,45,46].

On the other hand, sperm motility is essential for successful fertilization, so low sperm motility is highly associated with male infertility [47,48]. In fact, this defect has been the subject of research for years because it is frequently observed in andrology laboratories. In this sense, a retrospective study based on a large population reveals that about 82% of infertile men had impaired sperm motility [48]. Asthenozoospermia (AS), characterized by normal concentrations of spermatozoa (>15 million spermatozoa mL^−1^) and sperm progressive motility <32% [3], is one of the major causes of male infertility, which approximately accounts for 20% of infertility among men. The etiology of AS is varied and can be seen as a unique condition in isolated disease, associated with other sperm anomalies or as part of a syndromic association. In some cases, routine clinical examinations do not find clear causes, leading to so-called idiopathic asthenozoospermia [41]. Although the lower expression of several proteins might cause spermatozoa with poor motility, the molecular basis of AS is difficult to establish. Nonetheless, proteomic studies on asthenozoospermic individuals have increased in recent years, promoting the idea that the number of identified proteins related to sperm motility is rising [43].

For instance, in four different proteome analyses comparing sperm samples from asthenozoospermic vs. normozoospermic men, the altered expression of the HSPA2 protein was found. Interestingly, increased expression was observed in two studies [38,42], whereas HSPA2 expression decreased in the other two works [39,43]. Other chaperones HSPs (HSPA5, HSPA9, and HSPA1L) were also found downregulated in asthenozoospermic men [41,43]. Additionally, in a recent study comparing proteomes of high or low-motility human spermatozoa, HSPA1L and HSPA9 were also significantly decreased in low-motility spermatozoa [49]. Conversely, in other comparative sperm proteomics studies, the expression level of chaperones did not indicate significant differences [37,44,45,46]. It has to be mentioned that the lack of agreement or the opposite expression differences between the studies published may be due to several factors such as different quantitative technologies, different sample sources, low sample size, or even ethnic differences, among others. HSPs have a potential relationship with sperm quality, and they are important in normal sperm physiology [50], spermatogenesis, and sperm maturation [51,52,53], although the association between their altered expression and impaired motility is not yet fully understood. It is established that the role of the HSPs is to ensure the correct folding of proteins, their refolding of misfolded protein, and the orientation control of tagged proteins for subsequent degradation [54]. Thus, reduced HSPs expression might be associated with a decrease in sperm motility due to the accumulation of misfolded protein [55].

In addition, a higher level of triosephosphate isomerase (TPI), an extremely efficient metabolic enzyme in glycolysis and gluconeogenesis, is also associated with a reduced sperm motility phenotype [37,39,42]. Other proteins responsible for energy metabolism that could play an important role in spermatozoa motility maintenance are COX proteins (COX5B, COX6B, COX20, and COX41), which are involved in the oxidative phosphorylation (OXPHOS) pathway and showed lower levels in AS [38,42,43,45,46]. In conclusion, these are some examples that highlight the importance of glycolysis and OXPHOS as major metabolic pathways that provide energy to support human sperm motility.

Moreover, a reduction in sperm motility may be affected by other proteins, such as SEMG1 and SEMG2, which work as seminal plasma motility inhibitor proteins and are found up-regulated in asthenozoospermic men [37,38,46]. Another group of proteins with altered expression in spermatozoa with impaired motility involves different subunits of the proteasome such as PSMA3, PSMB3, PSMB4, PSMB5, PSMB6, PSMC2, PSMC6, and PSMD11 [37,38,39,42,45,49]. The proteasome plays a key role in the formation of condensed spermatozoa because it mediates the protein turnover of ubiquitinated proteins during spermatogenesis, when many proteins and organelles are degraded [55]. So, defects in the proteasome system might lead to the accumulation of ubiquitinated molecules and be related to sperm motility [39]. It is worth noting that SEMG1 and PSMB5 are also downregulated in a proteomic study that compares the proteomic profiles of human sperm samples that had or had not achieved a previous pregnancy via ART [56]. In addition, low levels of the major cytoskeleton components in spermatozoa flagella such as tektins (TEK1, TEK4, and TEK5) [39,41,42], outer dense fibers (ODF2) [41,43,49], or tubulin proteins (TUBB2C, TUBB2B, and TUBA3C) [39,43,49] are also associated with reduced sperm motility in comparative proteomics studies. On the other hand, altered levels of another protein that plays a role in the movement and structural organization of cells, such as CLU, have been found. CLU expression is decreased in some analyses [40,49], whereas it is increased in others [38,43,46].

Proteomics is also a current methodology used to study variations in sperm proteins that are altered in some disorders. For example, the SPEF2 protein is widely expressed in cilia-related organs such as the lung, spleen, trachea, brain, and testis [57], and its encoding gene is involved in a genetically heterogeneous disorder such as the so-called multiple morphological abnormalities of the sperm flagella (MMAF) [58]. Moreover, spermiogenesis failure by a deficiency in SPEF2 causes severe asthenoteratozoospermia, characterized by reduced sperm motility and abnormal sperm morphology [58]. Recently, an MS-proteomic analysis of human spermatozoa from three individuals with SPEF2 mutations compared with normal controls showed that this protein regulates the expression of various proteins involved in the flagellar assembly with which it interacts [59]. This methodology allows one to understand the protein networks from the whole sperm proteome, being especially useful in the study of sperm tail development since sperm flagellum is composed of more than 1000 proteins in the case of humans [60].

Human sperm cryopreservation plays an important role in assisted reproductive technology for male fertility preservation and the treatment of infertile couples. In this regard, proteomics approaches have also been useful to study the pathogenesis of sperm cryo-damage during the process of cryopreservation, comparing the proteomic differences between fresh and cryopreserved human sperm [61,62]. Fu’s lab, using MS and a novel proteomics technology named data-independent acquisition (DIA), identified 174 proteins significantly deregulated, including four enzymes involved in glycolysis (GPI, LDHB, ADH5, and PGAM1) and other proteins related to propanoate, glyoxylate, pyruvate, and dicarboxylate metabolism and gluconeogenesis [62]. Five years before, another proteomic analysis had found that 37% of the proteins involved in the metabolism are differentially expressed between freeze-thawed and fresh sperm samples [61]. So, both studies, although using different proteomic strategies, conclude that metabolic pathways play an important role during sperm cryo-preservation. Interestingly, phosphoglycerate mutase proteins, PGAM1 and PGAM2 [46] and the glucose 6-phosphate isomerase (GPI), evaluated by proteomic approaches, are also found to be significantly decreased in AS [42,46]. Furthermore, the supplement with the product of GPI, fructose-6-phosphate, significantly promotes human spermatozoa motility in vitro [46]. So, it can be postulated that during spermatozoa cryopreservation, when a marked reduction in sperm motility occurs, the supplement with fructose-6-phosphate could also help to recuperate the rates of spermatozoa motility.

Altogether, there are a set of proteins related to sperm quality in the literature. However, the internal relationship and the mechanisms underlying abnormal protein expressions and defective sperm function are not clear yet [49].

## 3. Phosphoproteomics Technique in Male Fertility

### 3.1. Phosphorylation as Post-Translational Modification of Sperm Proteins

Defects in PTMs have been linked to numerous human diseases and disorders, so the importance of PTMs in maintaining normal cellular states is essential [63]. Hence, previous and emerging data indicate that some male reproduction diseases, including the failure of sperm motility, arise through the deregulation of PTMs in spermatozoa. Despite that, more than 431 reversible and irreversible PTM mechanisms exist in the cell [64], and we know that protein phosphorylation affects an estimated one-third of all cellular proteins [65], with most proteins phosphorylated at one or more sites in a mammalian cell [66]. However, we know only a small subset of the in vivo phosphorylation sites described. Most studies have focused on Ser, Thr, and Tyr phosphorylation (canonical phosphorylation), but there are other amino acid residues that are less common, including His, Lys, Arg, Asp, Glu, and Cys, that can also be phosphorylated (noncanonical phosphorylation) [67]. This variability of possibilities further complicates the effort in studying protein phosphorylation, whose consequences may affect its activation status [68].

Nonetheless, protein phosphorylation is not permanent due to the activity of phosphatases [69]. Consequently, the deregulation of kinases and phosphatases pathways is linked to many diseases, including infertility. So, deciphering the molecular elements that determine the biochemical balance of phosphorylation/dephosphorylation is essential for correct reproductive function.

### 3.2. Phosphoproteomics Technique

Phosphoproteomics is a large-scale analysis that identifies and quantifies the phosphorylated proteins in addition to the mapping of the phosphorylation sites in a complex biological sample using MS [70]. Briefly, an MS-based phosphoproteomics study on the role of in vivo phosphorylation in sperm physiology starts with isolating sperm cells from the seminal plasma and other cells coexisting in semen by swim-up procedure, density centrifugation, or different techniques. The purity of the sperm preparation and the removal of interfering compounds are critical steps in the process because any minor contamination could result in a false-positive identification [71]. Sperm proteins are then extracted and protein mixtures are digested with a specific protease, typically trypsin.

Once proteins are extracted, carrying out a phosphopeptides enrichment procedure before experimental analysis is necessary, given that, for example, almost 30% of all human proteins may be phosphorylated and that each phosphoprotein may exist as multiple phospho-isoforms with different relative abundances and stoichiometries [70]. This procedure allows/has its purpose the characterization from low femtomole level phosphorylated proteins and the improvement of selectivity by reducing the unspecific binding of non-phosphorylated peptides [72].

Among the wide selection of methodologies developed for phosphopeptides enrichment, the most extensively used in the study of sperm cells is immobilized metal ion affinity chromatography (IMAC) [17], which is based upon the affinity that phosphate exhibits towards immobilized metal ions and forms relatively stable complexes with these. So, the nature of the chromatographic stationary phase is of extreme importance [73]. Accordingly, titanium dioxide (TiO2) resin has been one of the most widespread methods for phosphopeptides enrichment from complex biological samples because it has a very high affinity for phosphopeptides, is extremely tolerant towards most buffers used in biological experiments, and is optimal for large-scale phosphoproteomics studies [74].

Later, the sperm phosphopeptides are detected using both conventional and advanced proteomic techniques. Two-dimensional (2D) gel electrophoresis separates sperm proteins based on peptides’ isoelectric focusing properties and molecular weight. A modified version named difference gel electrophoresis (DIGE) identifies differentially expressed proteins (DEPs) [75]. The analysis of advanced high-throughput techniques such as MALDI-TOF (matrix-assisted laser desorption/ionization time-of-flight) and LC-MS/MS (liquid chromatography-tandem mass spectrometry) detect low abundance peptides present in a sample with low protein concentration. Therefore, they overcome the limitations of conventional proteomics techniques. In addition, advances in chromatography techniques such as nano HPLC (high-performance liquid chromatography) or UPLC (ultra-performance liquid chromatography) methods enable the decrease of the internal diameter of the LC column to analyze low amounts of a sample with none or very low dilution and with increased sensitivity, which allows for higher sample throughput [73]. Those aspects are fundamental in phosphoproteomics studies.

In addition, MS offers numerous advantages for studying protein phosphorylation, enabling its quantitative, sensitive, and site-specific measure [76]. MS-based quantification strategies rely on light/heavy peptide intensities and can be divided into label-based and label-free approaches. Label-based quantitation methods utilize stable isotope labels by chemical, metabolic, or proteolytic labeling strategies. These are incorporated within the peptides, introducing an expectable mass difference within the two or more experimental conditions. The quantitation is based on comparing the peak intensity ratio of the labeled peptide pairs [77]. In contrast, label-free quantitation compares both relative and absolute protein quantity by utilizing signal intensity and spectral counting of the same peptide [77]. It is also one of the methods of choice in human sperm phosphoproteome studies [27,31] and has gained more acceptance because it shows the highest proteome coverage and is cost-efficient without adding additional steps to labeling samples with alternative differential mass tags [78].

**Figure 1:**
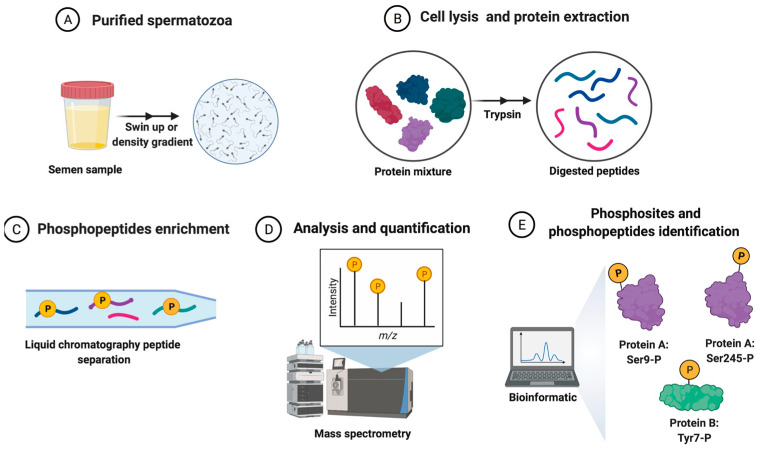
The general workflow of the quantitative phosphoproteomics strategy for human spermatozoa samples analysis from sperm donors. (**A**) Purified sperm cells from semen samples (spermatozoa isolated from other cells and the seminal plasma). (**B**) Sperm protein extraction and digestion after cellular lysis. (**C**) Phosphopeptides enrichment and peptide separation. (**D**) The analysis and quantification of peptides. (**E**) The collection and analysis of sperm phosphoproteome data by bioinformatics tools.

Finally, mass spectral data interpretation is carried out using the different platforms, databases, and software programs available, which allow for the identification and quantification of the assignments of peptides and proteins that make up the sperm phosphoproteome. Bioinformatics methods are indispensable for proteomics-based studies and are helping scientists to interpret the integration of large datasets from proteomics studies [79]. The specific workflow involving the processing of semen samples for sperm phosphoproteomics analysis is shown in Figure 1.

Furthermore, phosphoproteomics, in combination with these high-throughput techniques, is one of the most potent techniques nowadays for the global analysis of signaling networks in defined biological systems [80], such as human spermatozoa [27,31].

## 4. Phosphoproteomics and Spermatogenesis

As mentioned before, phosphorylation of sperm proteins is linked with male fertility because this PTM is extremely important in all stages of sperm cell development, being essential for sperm differentiation, maturation, and function [17]. To date, it is the most extensively studied PTM in mammalian spermatozoa, as previously mentioned. Besides, spermatozoa are an excellent cell model for proteomic analysis because they are purified in large numbers and are reliably and robustly driven into different functional states using various incubation media or validated pharmacological manipulations [81]. In fact, in the study of male reproduction, there is research identifying phosphoproteins and their phosphorylated sites through differential phosphoproteomics analysis of sperm cells from infertile men or under experimental or physiological conditions [18,24,29,31,32]. To include a summary of the use of phosphoproteomics studies carried out in human spermatozoa to study sperm biological processes, Table 1 is incorporated.

Defects in spermatogenesis are the most common factors for male infertility [4]. In this sense, phosphoproteomics tools are helpful to understand the origin of different causes of infertility because phosphoregulation is highly active during sperm differentiation [21,82,83]. Thanks to the technology for analyzing kinase-substrate relations (KSRs) in coordination with the exploration of the phosphoproteome, a pattern of consistently high activity for many kinases has been elucidated during spermatogenesis in mice, including the MAPKs, CDKs, and especially the POLO-like kinases (PLKs) [83]. In the same way, to decipher the most relevant signaling pathways during the development of male gametes in the human testes, Castillo and coworkers performed global phosphoproteomics on human testicular tissue with full spermatogenesis using a TiO2 method coupled to MS [21]. They identified 2661 proteins, and 174 of them were different phosphorylated kinases covering 32% of the human kinome, including MAPK1, MAPK3, CDK12, CDK13, and PAK4. Curiously, unlike in mice, PLKs are not among the most active kinases regulating human spermatogenesis [21]. In the same year, using proteomics and phosphoproteomics analyses of mouse testes, Wei’s lab suggested that WIP1 phosphatase is involved in maintaining the integrity of the blood–testicular barrier [84]. Therefore, as this organ is essential for spermatogenesis to progress correctly, its alteration results in male subfertility or infertility [84]. Besides, this phosphatase WIP1 seems to be an important regulator of global heterochromatin silencing and is critical in maintaining genome integrity [85], a basic regulatory mechanism for spermatozoa function. Although there are no studies that associate the role of WIP1 with male fertility in humans, this link has been observed previously in the mouse testis phosphoproteome [83].

During mammalian spermiogenesis, the last phase of the spermatogenesis, haploid round spermatids are differentiated into spermatozoa undergoing remarkable morphological changes, chromatin condensation, the biogenesis of the acrosome, the migration of mitochondria to the intermediate piece, and flagellum formation. A large-scale phosphoproteome analysis performed from purified mouse spermatids undergoing spermiogenesis described 735 testis-specific proteins phosphorylated and expressed at high levels. These phosphoproteins are implied in histone modifications and chromosome and cilium organization [22]. Nonetheless, defects at any level of the spermatogenesis, where protein phosphorylation is essential, would be inevitably associated with fertility impairments (such as azoospermia, teratozoospermia, oligozoospermia, or asthenozoospermia).

Evidence indicates that correct epididymal sperm maturation also requires protein phosphorylation and dephosphorylation events in spermatozoa. This epididymal process takes approximately two weeks in humans [86] and is necessary for generating fertile spermatozoa. When the spermatozoa leave the testes, they are immotile, and it is during their transit and storage through the epididymis when they acquire progressive motility and functional capacity for their interaction with the oocyte. During epididymal sperm maturation, besides phosphorylation, ubiquitination, a frequent PTM in regulating many sperm biological processes, is important as it eliminates defective spermatozoa (mostly with defects in morphology) by phagocytosis [87]. Interestingly, more and more scientific evidence shows that many proteins have different types of PTM simultaneously, which all together help to regulate protein stability and activity. The imbalance of theses PTMs’ crosstalk may be highly associated with male infertility; however, there are few studies about multiple PTMs co-occurring in sperm proteins (except for histones and protamines) [88]. Zhang and collaborators recently combined the phosphoproteome with the ubiquitylome to study the physiological mechanisms underlying sperm maturation in epididymal spermatozoa of buffalo [23]. Since a few years ago, sperm ubiquitination has been a marker of defective spermatozoon in humans [89]. For these reasons, mistakes in the post-testicular maturation context will affect male fertility. Based on these strategies, new biomarkers of sperm quality or semen abnormalities will help establish a precise landscape of PTMs that features high-quality spermatozoa.

## 5. Phosphoproteomics and Sperm Motility

In male reproduction, the use of phosphoproteomics is mainly focused on unraveling the molecular mechanisms underlying the regulation of sperm motility [71]. The first evidence about the control of sperm motility by processes that include the regulation of protein phosphorylation was described in dog spermatozoa in 1982 [90]. Later, two protein kinases, PI3K and AKAP3, were demonstrated to be involved in the phosphoregulation of human sperm motility [91]. On the other hand, as mentioned before, the phosphorylation status of a protein depends on the opposing activities of protein kinases and phosphatases. Accordingly, immotile mammalian spermatozoa contain higher activity levels of serine/threonine phosphatase 1 isoform gamma 2 (PP1γ2) compared with motile ones [92]. Moreover, the inhibition of PP1γ2 causes motility initiation in immature spermatozoa, whereas it leads to motility stimulation and changes in flagellar beat parameters in mature spermatozoa [93], indicating that protein phosphatases also regulate flagellar motility. Nowadays, high-throughput techniques are used to precisely decipher the phosphoproteome with functional importance for sperm motility, and the phosphoproteomic profiles of spermatozoa in different functional states (uncapacitated vs. capacitated; normal vs. defective, high vs. low-mobility).

Focusing on phosphorylation at tyrosine residues, phosphotyrosine-containing proteins are present in the spermatozoa of different species. For decades, it has been known that an increase in the protein tyrosine phosphorylation of spermatozoa plays a critical role in regulating sperm motility [94], especially in those processes related to hyperactivated motility. Moreover, deficiencies in the tyrosine-phosphorylated proteins of the sperm tail are associated with AS in human [95]. Later studies have focused on the differential analysis of the phosphorylation status of human sperm proteins by large-scale phosphoproteomics techniques in spermatozoa from healthy and asthenozoospermic donors [18,29] and from two sperm populations with different sperm motility degrees isolated from normozoospermic healthy donors [31]. Despite the different strategies of phosphoproteomics used, changes in sperm motility patterns correlate with the differential phosphorylation of proteins. Using 2D-gel electrophoresis MALDI-TOF/MS, the first study identified 12 proteins exhibiting differential phosphorylation. There was a relatively lower phosphorylation level in asthenozoospermic spermatozoa for 10 proteins, while in 2 of them, the level was higher [18]. In the same way, comparing healthy and asthenozoospermic semen, Parte and collaborators, using IMAC nano UPLC/MS, detected 66 sperm phosphoproteins with altered expression (39 were up and 27 were hypophosphorylated) in asthenozoospermic donors [29].

**Figure 2:**
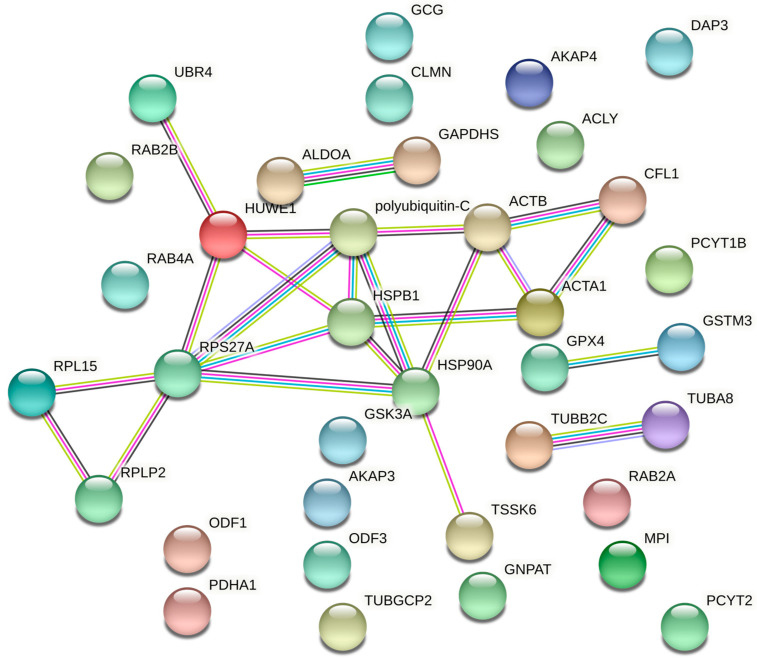
The protein–protein interactions (PPI) network of some phosphoproteins identified by comparative phosphoproteomics studies between human spermatozoa with different levels of motility. PPI are generated by the STRING database (http://string-db.org/, accessed on 14 April 2022). Bubbles show phosphoproteins involved in sperm motility, and lines represent both functional and physical protein associations. The colors of the lines indicate the type of interaction evidenced from different sources: databases (blue), protein homology (grey), high-throughput experiments (pink), co-expression experiments (black), and prior knowledge from research publications (yellow). The absence of a line indicates that no interaction has been detected. Only those interactions with a high confidence interaction score (score ≥0.7 according to STRING indications) are shown.

Moreover, a recent study of human spermatozoa using nano HPLC-MS/MS triple TOF confirms that the sperm proteins phosphorylation level is involved in sperm motility regulation. In fact, human sperm subpopulations with low and high motility statistically differ in up to 119 sperm phosphoproteins [31]. The constructed networks by the STRING database of protein–protein interactions of human spermatozoa from the phosphoproteins identified by the studies of Chan et al. (2009), Parte et al. (2012), and Martin-Hidalgo et al. (2020) (Table 1) and mentioned in the following paragraphs are shown in Figure 2. Combining overlapping data in these studies focused on human sperm motility, the most differentially expressed phosphoproteins were mainly involved in sperm metabolism. For example, GSTM3, a protein important in glutathione metabolism and cellular detoxification, was hypophosphorylated in asthenozoospermic donors [18,29]. Moreover, Ras-related proteins such as RAB2A, RAB2B, or RAB4A, which are involved in vesicle trafficking and ribosomal proteins such as RPLP2, RPL15, or DAP3, involved in the metabolism of proteins, also showed differential protein phosphorylation [18,29,31]. In addition, those proteins, related to the degradation processes in the protein ubiquitination pathway, such as the ubiquitin-protein ligases, UBR4 and HUWE1; polyubiquitin-C; and the ribonucleoprotein ubiquitin-40S ribosomal proteins S27a and RPS27A, were found to be hyperphosphorylated in low-mobility spermatozoa [29,31]. Other phosphoproteins involved in carbohydrate metabolism and energy production, such as pro-glucagon, fructose-bisphosphate aldolase A (ALDOA), glyceraldehyde-3-phosphate dehydrogenase-S (GAPDHS), mannose-6-phosphate isomerase (MPI), or the subunit alpha of the pyruvate dehydrogenase E1 component (PDHA1), also showed altered phosphorylation levels between two sperm subpopulations with very different motility degrees [18,29,31]. Specifically, MPI and PDHA1 were hypophosphorylated in spermatozoa with poor motility patterns [31], while the phosphorylation level of pro-glucagon, ALDOA, and GAPDHS was higher [18,29]. Interestingly, mice sperm lacking GAPDHS show deficient phosphorylation levels of sperm protein phosphatase 1 (PP1) accompanied by defects in sperm motility and male fertility [96]. However, changes in sperm motility do not correlate with differential PP1 phosphorylation in these phosphoproteomic studies. Regarding lipids metabolism, phosphoproteins such as ATP-citrate synthase (ACLY), dihydroxyacetone phosphate acyltransferase (GNPAT), ethanolamine-phosphate cytidylyltransferase (PCYT2), choline-phosphate cytidylyltransferase B (PCYT1B), or phospholipid hydroperoxide glutathione peroxidase (GPX4) were found to be altered according to the motility status of each spermatozoon [29,31]. While the phosphorylation levels of ACLY, GNPAT, PCYT2, and PCYT1B were increased, GPX4 was hypophosphorylated in low-mobility spermatozoa. These results highlight the importance of metabolism in different motility patterns in human spermatozoa. In fact, up to 40% of human proteins that are hyperphosphorylated in low-mobility spermatozoa are involved in metabolism [31]. This interesting finding is consistent with the differences observed by the proteomic approach in the expression levels of sperm proteins between healthy and asthenozoospermic individuals [45,46].

Other matching human phosphoproteins that are also found at altered levels in spermatozoa with impaired motility include those associated with flagellum assembly and motility [18,29,31]. The flagellum, a fundamental structure for spermatozoa motility, requires a specific organization of microtubules. The protein components of the tubulin superfamily (TUBB2C, TUBA8, or TUBGCP2, among others), together with actin and acting-binding proteins such as ACTA1, ACTB, CFL1, or CLMN, play an important role in the assembly of microtubules and consequently in sperm motility. These cytoskeletal proteins showed altered phosphorylation levels in AS vs. normal donors and high vs. low-mobility human spermatozoa [18,29,31]. In such a way, four of them were hyperphosphorylated (TUBB2C, ACTB, CFL1, and CLMN) in spermatozoa with poor motility [29,31]. Furthermore, lower phosphorylation levels are important to regulate flagellum functions in proteins such as components of the fibrous sheath such as AKAP3, AKAP4, or FSIP2, and elements of the outer dense fibers, such as ODF1 and ODF3, located in the midpiece and principal piece of the tail spermatozoa. All of them are related to sperm motility showing hypophosphorylated levels in AS and low-mobility human spermatozoa [29,31]. These data agree with the significantly increased phosphorylation of AKAP3 and AKAP4 observed during hyperactivated motility (movement with high amplitude and asymmetric thrashing of the sperm tail) associated with capacitated human spermatozoa [24,27]. These two PKA-anchoring proteins, which mediate the PKA activity by localizing this kinase to specific cellular structures and organelles, are postulated to coordinate sperm capacitation events, including motility hyperactivation [97].

On the other hand, the proteins of the heat shock family HSP—HSPB1 and HSP90A—were hyperphosphorylated in low-mobility human spermatozoa [29,31]. These HSP mediate protein folding and signal transduction and prevent protein aggregation predominantly; their role in human sperm function and male fertility is not clear yet, although a potential correlation with sperm quality has been described [98].

Other phosphoproteomics techniques to study protein changes associated with sperm motility were applied during sperm cryopreservation, when a marked reduction in sperm motility after freezing and thawing occurs. Decreased motility parameters are the most significant phenotype of cryodamage. Indeed, the cutbacks of human sperm motility were between 25% and 75% of the total in an evaluation of freeze-thawed sperm samples relative to that of fresh sperm samples from the same normozoospermic donors [99]. The level of tyrosine phosphorylated proteins in the freeze-thawed group [61], as well as the number of several kinases required for sperm production and function, such as testis-specific serine/threonine-protein kinase 6 (TSSK6) [100], was higher than those in the fresh group. Recently, several phosphoproteomics studies have been performed to investigate the molecular differences between fresh and cryopreserved (post-thaw) human spermatozoa. Wang’s lab constructed a quantitative phosphoproteome to investigate the expression change of phosphorylated sites during sperm cryopreservation [32], and they identified glycogen synthase kinase 3A, GSK3A, as a key kinase that may play an important role in regulating human sperm motility. The A isoform of the serine threonine kinase GSK3 contains at least five phosphorylation sites, including phosphorylation at Ser21, which inhibits its kinase activity and negatively correlates with human sperm motility [31,101]. The regulatory role of GSK3A is also required for proper motility in other mammalian spermatozoa [102,103,104,105]. Therefore, low GSK3 activity (more phosphorylated at Ser21), together with PP1γ2 activity, might be a prerequisite for the optimum function of mammalian spermatozoa [93].

STRING analysis revealed 23 high-confidence PPI collected from direct (physical) and indirect (functional) associations between 18 of the 36 phosphoproteins involved in sperm motility that were mentioned in previous paragraphs. So, 50% of the phosphoproteins identified by comparative phosphoproteomics studies between human spermatozoa with different motility levels are associated with one another (Figure 2). Moreover, focusing on protein functions, up to 56% are involved in sperm metabolism. These studies provide important information about proteins or their molecular mechanisms associated with male infertility, related to low-mobility, overall. However, they did not provide any evidence on the spermatozoa phosphoproteomics based on the type of infertility. A recent study combining phosphoproteomics results with functional analysis in human spermatozoa to analyze the role of G-protein-coupled receptors (GPCRs) in spermatozoa physiology identified phosphorylation changes in sperm-specific proteins downstream of the kappa-opioid receptor, which modulates human sperm motility [30].

Despite the revelations of these studies, molecular characteristics associated with the ability to fertilize the oocyte are still poorly understood in spermatozoa. Up to now, few candidate phosphoproteins have been associated with AS or low sperm motility. Still, there are many uncharacterized phosphoproteins and undescribed phosphorylation residues in spermatozoa. Therefore, functional studies in the future should elucidate their importance in sperm physiology.

## 6. Phosphoproteomics and Sperm Capacitation

The other two crucial stages in male fertility are sperm capacitation and acrosomal reaction, with protein phosphorylation also being crucially involved. Sperm capacitation to acquire the ability to fertilize occurs during sperm transit through the female reproductive tract for a specific time [106,107]. This process can be achieved in vitro, and its physical manifestation is hyperactivated motility, a powerful and asymmetric sperm tail movement [108]. After ejaculation, mammalian spermatozoa move actively, but they must undergo capacitation to fertilize the ovum. This process requires a sequence of physiological and biochemical changes, including increases in tyrosine phosphorylation, mostly in tail proteins. The first study to identify proteins phosphorylated during human sperm capacitation by a proteomic technique used two-dimensional polyacrylamide gel electrophoresis (PAGE), anti-phosphotyrosine antibody labeling, and MS/MS [24]. In that study, during human capacitation, the tyrosine phosphorylation sites of AKAP3 and AKAP4 proteins were detected, which are the major structural component of sperm fibrous sheath [24]. Later, another quantitative phosphoproteomics analysis comparing uncapacitated vs. capacitated human spermatozoa supported these results about AKAPs using IMAC-TiO2 (LC)-MS/MS [27]. These mentioned functional assays to study signaling pathways during human sperm capacitation, where phosphoproteomics analysis was combined with a prediction of cellular kinase-substrate relationships, indicated that the insulin growth factor 1 receptor, IGF1R, is an enriched tyrosine phosphorylation kinase. This receptor interacts during the capacitation process with the up-regulated phosphorylation sites for AKAP3 or AKAP4 proteins. As a result, the phosphorylation levels of AKAP3 and AKAP4, which include six and two phosphotyrosine sites, respectively, are significantly increased during human capacitation [24,27]. This tyrosine phosphorylation pathway mediated by IGF1R is crucial for human sperm capacitation and hyperactivated motility [27].

## 7. Outlook

Definitely, phosphorylation, among the PTMs, plays a vital role in regulating sperm processes that are essential for fertilization. Therefore, all these reviewed data together are a strong indicator of the contributions of sperm phosphoproteins in the most important male reproductive functions. So, the end goal of using proteomics technologies in male reproduction is to generate relevant data to increase the knowledge and the discovery of noninvasive predictive biomarkers of the prognosis and diagnoses of male infertility.

The precise cause(s) of male infertility remains elusive, although the correlation between immotile or poorly motile spermatozoa and infertility is scientifically strong. PTMs by phosphorylation allow eukaryotic cells and spermatozoa to dynamically regulate their intracellular signal integration and physiological states. Up to now, there have been some candidate human sperm phosphoproteins associated with AS or low sperm motility, but many uncharacterized sperm phosphoproteins and undescribed phosphorylation sites still exist. Nonetheless, providing clarification through functional studies would provide unprecedented insights into the regulatory tasks of phosphorylation and the molecular networks that govern spermatozoa function. The application of global phosphoproteomics profiling technology in evaluating sperm quality associated with male infertility would allow for significant advances to be made in identifying male infertility biomarkers. Therefore, more studies will improve the application of this procedure and overcome the limitations, providing data that will contribute to new knowledge and abundant resources for the screening of these molecular biomarkers in correlation with sperm quality. In addition, phosphoproteomics has found differences in spermatozoa from a distinct breed of pigs presenting different sperm qualities [109]. This research, which elucidates the mechanisms of regulation of male reproduction in other mammals, might be extrapolated to humans. In line with this, future studies of phosphoproteomics in a larger population could help explain whether the different results observed in some proteomics studies can be attributed to biological variation in various ethnic groups, as Siva et al. (2010) has speculated. Hence, further investigations combined with other single-omics fields (such as genomics, epigenomics, transcriptomics, and metabolomics) or with multi-omics contributions (proteogenomics, proteotranscriptomics, or reproductomics) are necessary. Findings from new studies would broaden our understanding of the mechanisms underlying the role of protein phosphorylation in sperm motility and male fertility. They would offer a unique perspective for future research into male fertility, especially for repeated failures in ART linked to unknown infertility causes.

## Figures and Tables

**Table 1:** A summary of phosphoproteomics studies in human spermatozoa biological processes.

Biological Process	Study	Type of Samples	Sperm Preparation	Phosphoproteomic Method
Spermatogenesis	Castillo et al. 2019	Testicular tissue		LC-MS/MS
Sperm motility	Chan et al. 2009	Normozoospermic vs. asthenozoospermic spermatozoa	Percoll fractionation	2DE-MALDI-TOF MS
	Parte et al. 2012	Normozoospermic vs. asthenozoospermic spermatozoa	Washing	Nano UPLC-MS
	Martin-Hidalgo et al. 2020	High-mobility vs. low-mobility sperm subpopulations	PureSperm fractionation	Nano HPLC-MS/MS Triple TOF
Sperm capacitation	Ficarro et al. 2003	Capacitated vs. non-capacitated spermatozoa	Percoll fractionation	2DE-anti-phosphotyrosine Immunoblots MS/MS
	Wang et al. 2015	Capacitated vs. non-capacitated spermatozoa	Percoll fractionation	LC-MS/MS

## Data Availability

Not applicable.

## Patents

This research/review did not generate patents.
